# Allergic diseases and asthma in pregnancy, a secondary publication

**DOI:** 10.1186/s40413-017-0141-8

**Published:** 2017-03-02

**Authors:** Isabella Pali-Schöll, Jennifer Namazy, Erika Jensen-Jarolim

**Affiliations:** 1 Comparative Medicine, The Interuniversity Messerli Research Institute of the University of Veterinary Medicine Vienna, Medical University Vienna and University Vienna, Veterinärplatz 1, 1210 Vienna, Austria; 2 Scripps Clinic, 7565 Mission Valley Rd Ste 200, San Diego, CA 92108 USA; 3 Institute of Pathophysiology and Allergy Research, Center of Physiology, Pathophysiology and Immunology, Medical University Vienna, Vienna, Austria; 4 AllergyCare, Allergy Diagnosis and Study Center Vienna, Vienna, Austria

**Keywords:** Allergy, Atopy, Newborn, Pregnancy, Prevention

## Abstract

Every fifth pregnant woman is affected by allergies, especially rhinitis and asthma. Allergic symptoms existing before pregnancy may be either attenuated, or equally often promoted through pregnancy. Optimal allergy and asthma diagnosis and management during pregnancy is vital to ensure the welfare of mother and baby.

For allergy diagnosis in pregnancy, preferentially anamnestic investigation as well as in vitro testing should be applied, whereas skin testing or provocation tests should be postponed until after birth. Pregnant women with confirmed allergy should avoid exposure to, or consumption of the offending allergen. Allergen immunotherapy should not be initiated during pregnancy. In patients on immunotherapy since before pregnancy, maintenance treatment may be continued, but the allergen dose should not be increased further. Applicable medications for asthma, rhinitis or skin symptoms in pregnancy are discussed and listed.

In conclusion, i) allergies in pregnancy should preferentially be diagnosed in vitro; ii) AIT may be continued, but not started, and symptomatic medications must be carefully selected; iii) management of asthma and allergic diseases is important during pregnancy for welfare of mother and child.

## Background

**Fig. 1:**
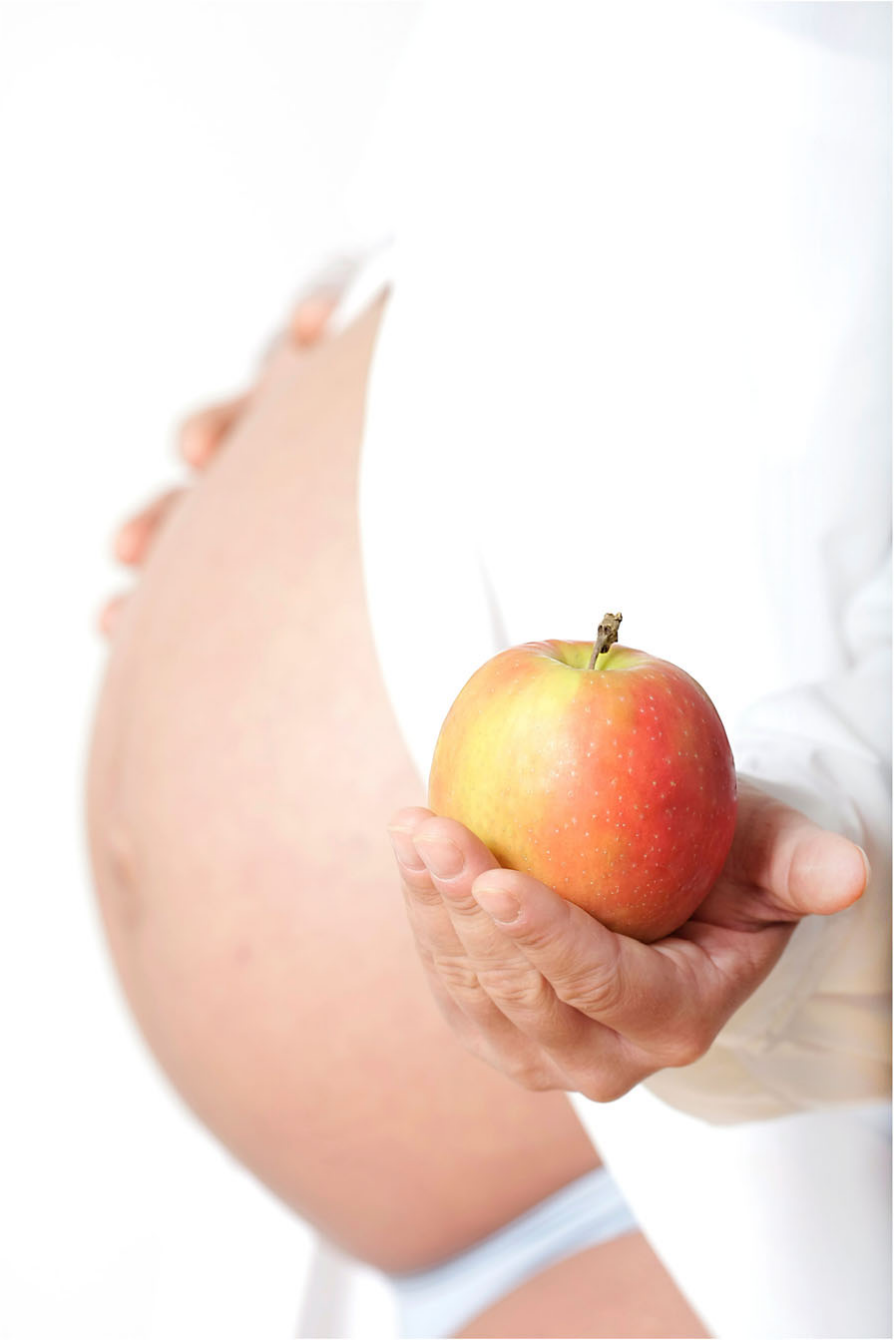
Management of asthma and allergic diseases are decisive during pregnancy for welfare of mother and child. (Fotolia.com©Reicher)

In the USA, about 18–30% of women in the childbearing age suffer from allergic diseases, and around 20% of pregnant women are affected by allergies, especially rhinitis and asthma. These two conditions often are present in the same patient (reviewed in [1, 2]). Other medical conditions that often complicate pregnancy include allergic conjunctivitis, acute urticaria, anaphylaxis, food allergy and drug allergy. Optimal management of these disorders during pregnancy is vital to ensure the welfare of the mother and the baby (Fig. 1)﻿. In this review, we focus on the current recommendations for diagnosis, management and therapy for allergic diseases and asthma in women during pregnancy and/or lactation, as well as on risk factors and preventive measures for mothers and their children.

### Diagnosis of allergy during pregnancy

The diagnosis of allergy in pregnant women should focus on a detailed medical history and symptom analysis. For diagnosis, (i) a diary of allergy symptoms and (ii) avoidance of suspected allergens accompanied by monitoring of changes of allergic symptoms may be helpful. It has to be emphasized that it is important not to put the mother on a rigid elimination diet for diagnosis of food allergy, as this could negatively influence the nutritional status of both the mother and the growing infant.

In vitro diagnostic tools such as serologic tests for allergen-specific IgE, e.g. ImmunoCAP or radioallergosorbent test (RAST), or the lymphocyte transformation test for type IV allergy diagnosis are preferred to skin and provocation tests, which should be postponed until after birth because of possible, though rare, anaphylactic reactions [3]. The same applies to food and other challenge tests. Despite the fact that there are no harmful effects of patch testing during pregnancy or lactation known, most physicians deter testing as general precaution, and furthermore because test results can interfere with immunological changes due to pregnancy [4].

### Management of allergic diseases during pregnancy

Mothers with allergy should avoid exposure to, consumption of and contact with diagnosed specific allergens. Patients should also especially avoid the inhalation of any potent triggers for asthma, such as animal dander, house dust, tobacco smoke and irritating pollutants.

Allergen immunotherapy (AIT, SIT, SLIT should ideally not be initiated during pregnancy because of the risk of systemic reactions. However, the initiation of immunotherapy can be considered in pregnant patients for clinical high-risk indication like anaphylaxis caused by *Hymenoptera* (insect venom) hypersensitivity. For patients who were already on immunotherapy prior to the pregnancy, maintenance treatment may be continued safely during pregnancy [5]. The allergen dose should not be increased during pregnancy. If pregnancy occurs while the patient is in the build-up phase of immunotherapy and on a low dose, which probably is not therapeutic, immunotherapy could also be discontinued [6].

Newer studies indicate that allergen immunotherapy is not only improving the disease in the pregnant patient, but that this treatment might also prevent allergic sensitization in the child. However, more studies are needed to confirm the effect of allergen immunotherapy during pregnancy on the development of sensitization in the child [7].

#### Medication for asthma and allergy in pregnancy

The ideal situation during pregnancy is “no pharmacologic therapy”, especially during the first trimester. However, in practice, medications must be considered for pregnant patients with medical disorders, based on a thorough appreciation of the potential deleterious effects of untreated disease in the mother, and also potential harm for the unborn [8]. For instance, women suffering from asthma require drug therapy during pregnancy to prevent life threatening episodes to the mother, as asthma exacerbations during pregnancy have been linked to a higher risk of pre-eclampsia, gestational diabetes, placental abruption and placenta praevia [9]..

Most of the existing data regarding asthma and allergy medications during pregnancy have not demonstrated adverse effects (Table 1), even though in infants of corticosteroid-treated mothers an increased risk of oral clefts, preeclampsia, preterm birth, and lower birth weight have been reported. Many of the case controls, which showed the association between oral corticosteroid and oral clefts did not provide information on dose, duration or indication. Other studies, which have demonstrated association with OCS and preterm delivery and low birth weight, have been linked with higher doses for longer periods. For example in Bracken’s study, which showed an association with OCS use and preeclampsia, the subjects were on OCS for the duration of pregnancy [10]. However, the potential side effects of any drug must be balanced against the risks to the mother or the infant of suffering from inadequately treated disease.

**Table 1:** Recommendations for treatment of asthma and allergies in pregnancy

Drug	Safety Data
Common asthma medications and safety data	
Inhaled bronchodilators (e.g. Albuterol, Formoterol and Salmeterol)	Human data generally reassuring for short acting and long-acting bronchodilators
Theophylline	Reassuring human data; serum levels must be monitored very closely to avoid toxicity
Systemic corticosteroids	Human data from smaller case control studies show increase in oral clefts. Larger prospective studies show increase in low birth weight, preterm birth, preeclampsia and intrauterine growth retardation.
Inhaled corticosteroids	Human data mainly reassuring. There may be an increased risk of malformations seen with higher doses.
Leukotriene Receptor Antagonist (e.g. Montelukast, Zafirlukast)	Human data are generally reassuring
5-Lipoxygenase-Inhibitor	Generally avoided during pregnancy due to the available less reassuring animal data.
Omalizumab	Increased risk of low birth weight and preterm birth; likely severity of asthma may confound to these observations.
Common allergic rhinitis medications and safety data	
Oral antihistamines (e.g. Azelastine, Cetirizine, Chlorpheniramine, Dexchlorpheniramine, Fexofenadine, Diphenhydramine, Hydroxyzine, Loratadine)	Human data are generally reassuring. Hydroxyzine should be used cautiously during first trimester based on animal data. Fexofenadine (an active metabolite of Terfenedine): no reports of increased congenital malformations, however, no epidemiologic studies in human pregnancy available.
Oral and Nasal Decongestants (e.g. Oxymetazoline, Phenylephrine, Phenylpropanolamine, Pseudoephedrine)	Should be avoided during pregnancy: Oxymetazoline has been associated with possible uteroplacental insufficiency at higher doses. Phenylephrine has been associated with clubfoot and eye/ear malformations. Phenylpropanolamine associated with congenital malformations, gastroschisis and ventricular septal defect. Pseudoephedrine associated with gastroschisis, hemifacial microsomia and small intestinal atresia in some case–control studies.
Intranasal Antihistamines (e.g. Azelastine, Olapatadine)	Animal studies are reassuring.
Intranasal Corticosteroids (e.g. Budesonide, Fluticasone, Triamcinolone, Mometasone)	Substantial reassuring data for inhaled corticosteroids. Risk of increased malformations at high dose, but severity of allergic rhinitis may be a confounding factor for these outcomes.

The recommendations from Table 1 have been reviewed in detail in [11, 12] (table modified from [13]). FDA categorization by letters has been removed for labeling of drugs used during pregnancy and lactation. New FDA regulations for labeling of mediations have been published in 2014 [14]

#### Treatment of asthma

Certain physiological changes occur normally during pregnancy, like increased tidal volume and minute ventilation, and decreased residual volume, functional residual capacity and diffusion capacity. These alterations are primarily the result of hormonal effects. The physiologically elevated position of the diaphragm and hyperventilation occurring in pregnancy further increase the risk of hypoxia. Preexisting asthma symptoms may worsen, improve, or remain unchanged during pregnancy. Each of these three possibilities is observed in about one third of cases. Optimal asthma treatment is crucial [8], as the risk of pre-eclampsia, premature birth, low birth weight, and maternal and neonatal hypoxia and morbidity posed by undertreated asthma may be greater than that from the use of oral steroids for the treatment of asthma.

Treatment of *acute* asthma is similar to that recommended for non-pregnant patients (reviewed in detail in [11, 12]), including inhaled beta2 agonists, oxygen (essential), and corticosteroids (oral or parenteral). It is also wise to add nebulized ipratropium bromide in patients who do not respond to beta2 agonists. Intravenous aminophylline is not generally recommended in the emergency management of acute asthma (because of its potentially harmful effects) but may be used in pregnant patients hospitalized for acute asthma (theophylline levels should be monitored). Intravenous magnesium sulfate may be beneficial in acute severe asthma as an adjunct to inhaled beta2 agonists and corticosteroids.

The goals of management of *chronic* asthma are the same as those for asthma in general, including prevention of severe exacerbations, improvement of quality of life (no interference with sleep or daily activities) and maintenance of normal lung function. The recommendations for medical treatment have been summarized by the Global Initiative for Asthma (GINA) working group including management of asthma during pregnancy [15]. A step-wise approach is suggested for medical treatment. Inhaled salbutamol is the preferred short-acting beta-agonist, with an outstanding safety profile, and among inhaled corticosteroids budesonide is preferred based on the available data. Salmeterol is the preferred agent when long-acting beta2 agonists are indicated in a pregnant woman as add-on treatment for persistent asthma. Leukotriene modifiers may be used as alternative add-on treatment: montelukast and zafirlukast are the preferred anti-leukotriene drugs. Zileuton in contrast, being the only leukotriene synthesis inhibitor, is not recommended in pregnancy due to its potential to cause abnormal liver function (FDA pregnancy category C).

Patients whose asthma is not controlled with maximal doses of bronchodilators and anti-inflammatory agents may need systemic corticosteroids. The lowest possible effective dose should be used. Patients must be monitored closely for potential adverse effects of corticosteroids, especially gestational diabetes, preeclampsia, and intrauterine growth retardation. Based on the available data, control of maternal asthma is essential to reduce the risk of perinatal complications. As pregnant women are hesitant about continuing asthma medications during pregnancy, asthma education is a critical component in the management of the pregnant asthmatic patient.

One of the treatment options for moderate to severe persistent allergic asthma is the recombinant DNA-derived humanized IgG1k monoclonal antibody omalizumab (Xolair®), which specifically binds to free human immunoglobulin E (IgE) in the blood. It currently has an FDA Category B classification based on reassuring animal studies and the expected limited placental passage in the first trimester due to the size of the molecule. We have established an ongoing registry with a target goal of enrolling 250 asthmatic women treated with omalizumab during pregnancy [16].

#### Treatment of rhinitis

Significant nasal symptoms occur in approximately 30% of pregnant women. Pregnancy-associated hormones have direct and indirect effects on nasal blood flow and mucous glands. The most common causes of nasal symptoms necessitating treatment during pregnancy are allergic rhinitis, *rhinitis medicamentosa*, sinusitis, and (non-allergic) vasomotor rhinitis. “Vasomotor rhinitis of pregnancy” or pregnancy rhinitis is a syndrome of nasal congestion and vasomotor instability, limited to the gestational period. Allergic rhinitis commonly co-exists with asthma. As with asthma, pre-existing allergic rhinitis can worsen, improve, or remain unchanged during pregnancy.

The general principles of treatment for pregnant women with allergic rhinitis [17, 18] –as with asthma- and do not differ from the step-wise approach recommended for treatment of non-pregnant women. The initial treatment steps are non-pharmacological and shall include avoidance of allergens and irritants, furthermore, nasal lavages with salty water solutions. The mainstays of pharmacological therapy for allergic rhinitis in non-pregnant as well as pregnant patients are antihistamines and intranasal glucocorticoids. No important differences in efficacy or safety appear to exist between the various intranasal glucocorticoid preparations. Most pregnant women who require antihistamines for allergic rhinitis are appropriately treated with a second generation agent, because these drugs are less sedating and have fewer cholinergic side effects compared with first generation agents. Among second generation antihistamines, loratadine (10 mg once daily) and cetirizine (10 mg once daily) may be considered the second generation antihistamines of choice in pregnancy.

For decongestant treatment, there are insufficient safety data. The narrowing of blood vessel due to this medication could have negative effects on the fetus, and furthermore, decongestant nasal sprays can cause addiction. These medications should therefore be avoided during pregnancy.

#### Treatment of anaphylaxis

The management of anaphylaxis during pregnancy [3] is similar to treatment of non-pregnant patients. The first step is to avoid the trigger of the anaphylactic reaction. For the treatment of anaphylaxis, epinephrine (adrenaline) should be promptly injected i.m. Adequate intravascular volume repletion and oxygenation are particularly important in the management of anaphylaxis during pregnancy to prevent both maternal and fetal complications. The pregnant hypotensive patient should be placed on her left side to prevent additional positional hypotension resulting from compression of the *vena cava inferior* by the gravid uterus, with her lower extremities elevated. Intravenous epinephrine may be required, despite its potential to cause decreased uteroplacental blood flow. Glucocorticoids should be administered early to patients with severe anaphylaxis. For laryngeal spasm, intubation and in rare cases tracheotomy may be necessary.

#### Treatment of atopic eczema/dermatitis

Gestational itchy dermatoses are relatively common, with eczema being diagnosed in 36 to 49% of all pregnancy dermatoses. Treatment of atopic dermatitis during pregnancy [19] should emphasize avoidance of triggering factors and reliance on topical treatment with emollients to nourish and re-establish the skin barrier. Topical corticosteroids are prescription-dependent first-line treatment, however, they should only be initiated when clinically indicated with the least potent effective preparations. Oral antihistamines (AH) may be required as systemic treatment. Short-term use of (sedating) first-generation antihistamines may be beneficial in the setting of sleep loss secondary to itch [20]. Chlorpheniramine and diphenhydramine are considered safe during the first trimester. However, also second-generation drugs are generally safe, and loratidine is the preferred second-generation antihistamine in pregnancy (reviewed in [19]). In general, AH should be used cautiously in the last month of pregnancy, because of possible withdrawal symptoms in the child, like poor feeding, diarrhea, irritability, or tremulousness, which can last up to 4 weeks after birth [21, 22]. Atopic dermatitis can additionally be managed with UV phototherapy (UVA, broadband UVA and UVB, or narrowband UVB).

#### Treatment of urticaria and angioedema

The pattern and causes of urticaria and angioedema in pregnancy are similar to those in non-pregnant patients. A unique form of urticaria associated with pregnancy (“pregnancy urticaria”, Pruritic urticarial papules and plaques of pregnancy PUPPP) mainly occurs in primigravida mothers in the last trimester [23, 24]. The first step in treatment of urticaria and angioedema [25] in pregnancy is identification and avoidance of causative factors. Antihistamines should be avoided if possible, but if required, the lowest dose of chlorpheniramine, loratadine, or cetirizine may be used.

### Risk factors for atopy

The causes of allergy in general and of specific sensitization in newborns in particular have not been completely determined yet. Besides the role of genetic predisposition, some factors have been identified that may either contribute to sensitization of the mother and to the subsequent transfer of a predisposition for allergy to the offspring, or that directly induce sensitization in the offspring, that manifests shortly after birth or at a young age (reviewed in [26]).

#### Family history of atopy/allergy

The degree of risk for atopy/allergy appears to be directly related to the family history of allergy and especially to maternal atopy. If neither parent is allergic, the chance for allergies in the child is about 5–16%. If one parent is allergic, the risk increases to 20–40% (father: 33%, mother: 45%), and if both are allergic, the risk is greater than 40–60% (if patients have the same allergy: 50–80%), especially for developing the same organ-specific symptoms [27].

#### Exposure to tobacco smoke

In a recent human study performed by parental questionnaires, exposure to smoke *in utero* or during infancy enhanced the risk for asthma and rhinitis primarily in early childhood, and the risk for eczema at later ages of the children [28]. In human blood samples, Th2 cytokines responsible for a predisposition toward allergy were elevated in the neonates only of mothers who had smoked during pregnancy. In addition, total and specific IgE levels, total eosinophil counts, incidence of airway disease and positive results on skin prick tests were also increased in children who were exposed to smoke either during pregnancy or in early childhood [29].

#### Alcohol consumption

Alcohol consumption by the mother during pregnancy is associated with higher total IgE levels in cord blood [30] and furthermore with an increased risk of atopic dermatitis in the child [31]. Apart from these atopy-associated negative effects, alcohol consumption should be avoided during pregnancy due to general health concerns (e.g. fetal alcohol syndrome).

#### Maternal diet

Recent research has focused on the role of several essential nutrients in the diet of the mother, like Vitamin D, zinc, folate and n-3 polyunsaturated fatty acids (PUFAs) [32]. Contrasting data exist on the effects of n-3 PUFA. On the one hand, a diet higher in n-6 polyunsaturated fatty acids (PUFAs) -as present, for example, in margarine and vegetable oils- seems to be more likely to induce eczema than n-3 PUFAs, which are found in fish. Accordingly, several observational studies show that a high intake of fish and oily fish during pregnancy results in a reduced incidence of allergy in the children (reviewed in [33]). On the other hand, a recent Cochrane systematic review revealed no evidence for supplementation of the mother with n-3 PUFA during pregnancy and lactation for prevention of allergy in the child [34].

Current evidence suggests a protective effect of maternal intake of vitamin D, vitamin E, or zinc for wheezing in childhood, but the data are not conclusive for an effect on asthma or other atopic conditions [35].

The effect of folate and folic acid supplementation is intensively discussed. Higher levels in maternal blood seem to be positively associated with atopic dermatitis in the offspring [36]. Controversially, recent studies and a systematic review found no association of prenatal folic acid supplementation and atopic diseases in children [37, 38]. Importantly, apart from the effects on atopy and allergy, sufficient levels of folic acid uptake by the mother before and during pregnancy have been shown to reduce the risk for neural tube defects [39].

Regarding allergenic food consumption during pregnancy and lactation, there has been extensive reviewing of data. According to the updated directive (No. 1169/2011, entered into application on 13 December 2014) of the Commission of European Communities, the 14 most allergenic foods have to be labeled on pre-packed food, and this declaration/information has also to be provided for non-pre-packed food [40]. These allergen sources are crustaceans, mollusks, fish, nuts, milk, egg, cereals containing gluten, peanuts, soybeans, sesame, mustard, celery, lupines, and the products of all these, as well as sulphur dioxide and sulphites. Some studies suggest that allergen exposure during pregnancy, lactation and early childhood may be necessary to induce tolerance [41]. Accordingly, avoidance of allergenic food by the mother, e.g. milk, egg and nuts during pregnancy, did not appear to lower the risk of sensitization in the child [42]. Moreover, a balanced diet prevents malnutrition of both mother and child.

#### Use of anti-acid medication

Changes of hormone levels during pregnancy and the growing volume of the fetus often lead to heartburn, reflux and abdominal pain in the mother. About 70% of pregnant women are affected by these symptoms during their last trimester and 50% of them are likely to take acid-suppressing medication. However, animal and human studies indicate that acid suppression and the resulting elevated pH in the stomach may lead to an increased risk of sensitization to food ([43, 44], reviewed in [45]) and drugs [46, 47]. This mechanism was recently also shown to be true for children aged 0–18 years with gastro-esophageal reflux disease, who were treated with gastric acid suppression medication [48]. Importantly, a sensitization of the mother induced by acid-suppression was shown to lead to an increased risk of food allergy in the newborn in a BALB/c mouse model [49]. Also in a database-link study of human patients, the positive correlation between acid suppression during pregnancy and increased risk for asthma in children was shown [50]. Although more studies are needed, pregnancy-associated reflux should probably be treated by non-pharmacological measures first (avoidance of large meals, sleeping with elevated upper body, not lying down after a meal, avoiding sweet and fatty food as well as alcohol and smoking). In general, during pregnancy and lactation, patients should avoid intake of any medication including non-prescription over-the-counter substances, unless recommended and closely monitored by a physician.

#### Insufficient exposure to environmental bacteria

The “hygiene hypothesis” states that low exposure of the mother during pregnancy and of the newborn in early life to environmental bacteria contributes to a Th2-biased immune response. This hypothesis has been confirmed by several experimental animal and epidemiological human studies, whereas details about the mechanism are still under investigation [51].

#### Cohabitation with pets

In a recent longitudinal study the effects of ownership of a wide range of pets from pregnancy to 7 years of age were investigated [52]. Whereas cat ownership was associated with lower, rabbit and rodent ownership was associated with a higher risk of wheezing. In that study, dog ownership in pregnancy was associated with wheezing in the newborn at the age of 6 months. However, in studies on urban children, especially dog exposure was a clear protective factor against asthma and allergic diseases, at least in children without family predisposition for allergies [53]. Dogs also seem to protect from atopic eczema [54, 55]. The discussion is, however, ongoing. For instance, recent recommendations for the prevention of food allergy and atopic eczema again contained the recommendation to avoid pets during gestation [56]. It is anticipated that an exchange of immunomodulatory allergens such as lipocalins takes place between pets and humans [57]. Reptiles and exotic pets were so far not investigated in any birth cohort studies, but potent allergens may be expected from their feeding animals [58].

## Conclusion

Diagnosis, preferably by in vitro testing and avoidance of skin and provocation testing, as well as management of asthma and allergic diseases are decisive during pregnancy for welfare of mother and child. The most important initial steps for allergic pregnant women are the avoidance of the offending allergen and the symptomatic asthma and rhinitis treatment to guarantee optimal oxygen supply, also of the unborn. Allergen-specific immunotherapy should not be initiated, but can without further dose increase be maintained during pregnancy and lactation.

## Abbreviations

AH: antihistamines
AR: allergic rhinitis
IFN-γ: interferon gamma
IL: interleukin
PUFA: polyunsaturated fatty acids
PUPPP: pruritic urticarial papules and plaques of pregnancy
RAST: radioallergosorbent test
Th2: T helper type 2
USA: United States of America
